# China’s First Demonstration of Cobalt-rich Manganese Crust Thickness Measurement in the Western Pacific with a Parametric Acoustic Probe

**DOI:** 10.3390/s19194300

**Published:** 2019-10-04

**Authors:** Feng Hong, Haihong Feng, Minyan Huang, Binxian Wang, Jingjie Xia

**Affiliations:** 1 Shanghai Acoustics Laboratory, Chinese Academy of Sciences, Shanghai 201805, China; 2 University of Chinese Academy of Sciences, Beijing 100049, China

**Keywords:** acoustic probe, acoustic parametric array, wavelet method, wavelet regression, acoustic thickness measurement, underwater acoustic measurement, cobalt-rich manganese crust

## Abstract

Cobalt-rich manganese crusts (CRCs) are important as a potential mineral source that could occur throughout the Pacific on seamounts, ridges, and plateaus. We built a prototype parametric acoustic probe to complete the task of *in-situ* thickness measurements to estimate the volumetric distribution of deep-sea mineral. The prototype is designed with dual-channels for receiving the primary and secondary signal, which lays a foundation for improving the thickness extraction algorithm. Considering that the signal quality is degraded by the system interference and ambient noise, some improvements to the algorithm are proposed by including the wavelet-based envelope extraction method and the adaptive estimation strategy based on the dual-channel information. Additionally, wavelet regression is applied to reduce the measuring noise assuming that the CRCs have local thickness invariability. The algorithm is suitable for the CRCs with the structure of the multilayers at the top surface and one single layer at the bottom surface. A laboratory experiment is performed to validate the effectiveness of the algorithm. The experiments carried out on the China Ocean 51th voyage in the Western Pacific Ocean on Aug 30, 2018, are described and the data obtained by using the *sit-on-bottom* stationary measurement are processed to validate the design of the prototype.

## 1. Introduction

Cobalt-rich manganese crusts (CRCs) are important as a mineral resource on the seafloor for copper, cobalt, nickel, platinum, manganese, thallium, tellurium, and other metals [1]. CRCs could occur throughout the Pacific on seamounts, ridges, and plateaus, where currents have kept the rocks, swept clean of sediments, at least intermittently, for millions of years [1]. Crusts form at water depths of about 400 to 4000 m, with the thickest and most Co-rich crusts occurring at depths of about 800 to 2500 m, which may vary on a regional scale. Gravity processes, sediment cover, submerged and emergent reefs, and currents control the distribution and thickness of crusts on seamounts [1,2,3,4].

Cobalt has many special properties and plays an important role in industry. For example, its properties allow for increasing chip performance, lead it to have a major place in the semiconductor manufacturing industry. As cobalt works better at higher densities, it may be called to also play a decisive role in the new and innovative artificial intelligence field. It is estimated that the resources of cobalt in the CRCs may be comparable with the land-based resources. Rising demand for minerals and metals, in tandem with the depletion of land-based resources, has led to a surge of interest in marine mineral resources [5]. However, due to political uncertainties regarding ownership of the oceans and problems associated with mapping and exploring techniques in deep-sea water, these resources have so far remained unexploited. To address the political problem, the International Seabed Authority (ISA) has entered into 15-year contracts for exploration for polymetallic nodules, polymetallic sulphides, and cobalt-rich ferromanganese crusts in the deep seabed with twenty-seven contractors including China Ocean Mineral Resources Research and Development Association (COMRA). On July 27, 2012, China is approved for the exclusive right to explore an initial area of up to 3000 square kilometers. The cobalt contract starts on April 29, 2014 and ends on April 28, 2029. Under the Regulations, over the first five years of the contract, one-third of this area is to be relinquished. Over the second five years of the contract, another one-third of this area is to be relinquished. Finally, only the last one-third of this area can be kept for exploration.

From the aspect of techniques, mapping and quantitatively estimating the volumetric distribution of these deposits needs to be done first, which is of interest to geologists, oceanographers, and industry [3]. As a key step of the assessment, the measuring of the thickness can be performed by various methods. Traditional methods of estimation, such as sampling from Remotely Operated Vehicles (ROV) [6], dredges or core drilling can obtain samples for detailed, pointwise measurements of thickness and composition. However, they fail to capture the continuous local variations of the deposits. An alternative is to employ the traditional acoustic instruments such as bathymetric multibeam, side-scan sonar imaging, and acoustic sub-bottom mapping. Unfortunately, one major limitation of the thickness measurement is the resolution, which seems essential in the CRCs measurements. The reasons behind this rely on two aspects, i.e., the wide variety of the CRCs distributions and the diversity of their thickness. Specifically, on one hand, the CRCs grow on a wide variety of substrates, and subsequently, the level of contrast can vary significantly between different regions [7,8]. Hence, if the acoustic device has a fixed transmitter power, the reflection may be too small to detect the thickness of CRCs at some spots, while significant side-scan happens at other spots. On the other hand, traditional devices good at some penetrating applications may not be capable of obtaining enough resolution to distinguish layers with a small thickness. In summary, considering that CRCs have extremely uneven distribution and thin geometrical features, it remains a challenging task to collect the necessary information to measure the thickness by using such general acoustic devices at the aspect of resolution or the penetrating capability.

To complete the task of *in-situ* thickness measurements of CRCs at depths up to 3000 m, another kind of device, i.e., the acoustic probe developed by the Institute of Industrial Science of the University of Tokyo, can significantly improve the spatial resolution [4,9]. The acoustic probe is developed based on the parametric array principle, which has a performance of better beam directivity, narrower beamwidth, better resolution, and sidelobes compared with traditional acoustic arrays [10,11,12]. The acoustic probe employs a high-power acoustic pulse, whose sub-surface reflections are recorded [3]. The thickness can be simply determined by the time delay between the top and bottom reflection if the sound velocity within the CRCs is assumed to be known. The thickness measurement has been proven feasible after the laboratory test and sea trial.

Motivated by their device, we built the improved version of the prototype acoustic probe, i.e., Programmable Phased Parametric Array Acoustic Probe 2017 (PPPAAP17), to collect and process the data both for the thickness measurement and for the material recognition in future. Compared with the acoustic probe mentioned above, our device has several highlights. On one hand, from the perspective of the system design, our prototype has the capability of adjusting the focal length by the control of the phased array. In addition to this, the receiving system of our prototype consists of dual-channel, with one channel for the primary signal and the other for the secondary signal, which lays the foundation for the algorithm to better identify the bottom and top surface even in the environment of the signal to noise ratio (SNR). On the other hand, to further improve the robustness of the thickness extraction algorithm, our prototype utilizes an effective and stable algorithm for thickness processing.

It is very common to measure the thickness of the target by using the reflected mechanical waves. Such methods have been widely used in multiple applications, such as geological exploration [13,14] and medical acoustics [15,16,17]. In this study, we emphasize on the work for improving the performance of the thickness measurements, considering that PPPAAP17 is a parametric array acoustic probe designed for the application of estimating the resources of CRCs. In practice, the necessary parameters, i.e., the sound velocity, the arrival time of the top surface, and the arrival time of the bottom surface, are calculated by processing the received signal that contains complicated context with the top-bottom peak detection. The reliability of the calculation is affected by noise in the recorded signal arising due to scattering, multi-path reflection, and seafloor features such as local inclusions inside the crust [5]. Hence, considering that the signal quality is degraded by the system interference and ambient noise, the dual-channel signal information and the inner connection between the large amounts of the recorded data are exploited. Some improvements are introduced to the processing algorithm, including the adaptive candidate selection strategy and the wavelet techniques [18,19]. For clarification, the key ideas are listed here:

- The envelope is extracted by using wavelet transformation based on the dual-channel information which gives better stability compared to the traditional Hilbert envelope extraction method.
- The determination of the arrival time of the top surface not only relies on an adaptive candidate selection strategy suitable for real-time processing logic but the auxiliary information of secondary channel, while that of the bottom surface relies on the adaptive candidate selection strategy, the geometric constraints, and the auxiliary information of the primary channel.
- The wavelet regression is applied to further reduce ambient noise. The reason behind this is the assumption that the CRCs have the local thickness invariability. The utilization of neighborhood information of the large amounts of recorded data can further improve the robustness.

In this paper, we pay attention to multiple aspects, i.e., system design, processing algorithm, the laboratory experiment, and the real experiment. For the remaining sections, the problem formation is presented firstly. Afterward, the system description which includes the hardware configuration and the array design is briefly presented. Next, the processing algorithm is elucidated. Finally, the processing and analysis of the experiments are demonstrated, and the comparisons are given to verify the effectiveness of the system and the algorithm.

## 2. System Description

The parametric acoustic probe is designed to perform accurate remote measurements of CRCs with thicknesses greater than 30 mm and less than 350 mm. To satisfy both the requirements of along-track resolution and the measuring range, the proper trade-off should be made. We have known that finer along-track resolution needs the shorter wavelength of the signal, whereas the stronger penetrating capability needs the longer wavelength of the signal considering the attenuation. The probe transmits a 1 MHz, high-frequency amplitude modulated signal to generate a narrow 100 kHz beam that penetrates the target, which could achieve a narrow beam and increase the range of operation of the system. An acoustic beam of frequency 100 kHz on its target has been found to provide a suitable balance between resolution and signal attenuation when measuring CRCs with thicknesses in the range of interest [20].

**Figure 1:**
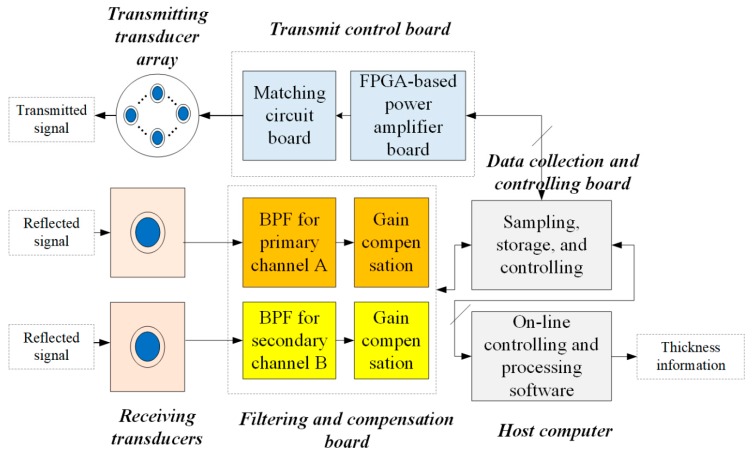
System description of PPPAAP17.

**Figure 2:**
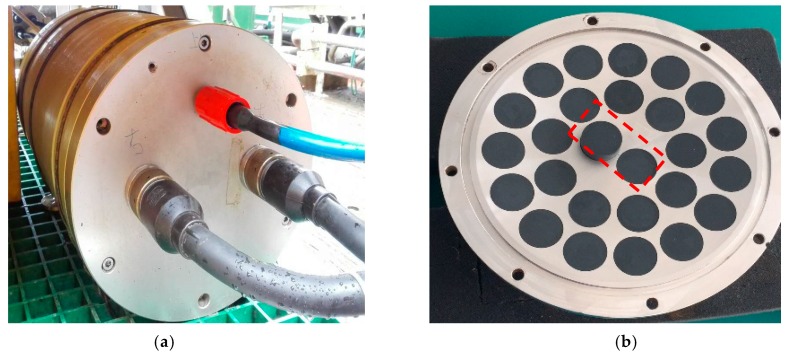
Hardware configuration: (**a**) The electronic processing unit that contains the transmit control board, the filtering and compensation board, and the data collection and controlling board; (**b**) The designed array with receiving transducers marked with the dashed box and the transmitting transducer array (outside the box).

As shown in Figure 1, PPPAAP17 mainly consists of two receiving transducers, two filtering and compensation boards, the transmitting transducer array, the transmit control board, the data collection and the controlling board, and the host computer. The real photo of the electronic processing unit is presented in Figure 2a, with the transmit control board, the filtering and compensation board, and the data collection and controlling board inside. When the system works, the host computer sends the transmitting parameter and command to the data collection and controlling board and the gain compensation parameters to the filtering and compensation board with the Ethernet protocol, respectively. Afterward, the data collection and controlling board will forward the information to the transmit control board to periodically drive the transmitting transducer array to fire. In the meantime, the sampling, storage, and controlling board is synchronized to receive the reflected signal from two separate receiving transducers, with one fed into the primary channel and the other fed into the secondary channel.

Considering that the probe, PPPAAP17, is designed for ROV currently, the size of the array should satisfy the requirements of both mass and volume. Note that the array has an impact on the across-track resolution since the interference caused by the scattering waves depends on the beamwidth. In other words, the cross-track resolution is finer if the beamwidth is narrower. We have performed many acoustic field simulations for multiple arrays. As shown in Figure 2b, the transmitting array design is optimal at the aspect of the penetrating capability and the across-track resolution.

At the aspect of receiving, the primary signal of high-frequency and the secondary parametric signal of low-frequency is obtained by feeding the signal received by the transducer centered at the array to two designed filters, which is marked in red. The two designed filters are used to improve the Signal to Noise Ratio (SNR) of the signal. The gain compensation part can be adjusted by the data collection and controlling board to obtain suitable signal amplitude. The filter and sampling parameters are listed in Table 1. Note that the secondary channel has two different modes with different Band Pass Filter (BPF) and sampling parameters. The sampled data are transferred and stored in a real-time way and processed by using the on-line controlling and processing software to obtain thickness information with an effective algorithm.

## 3. Processing Algorithm

**Figure 3:**
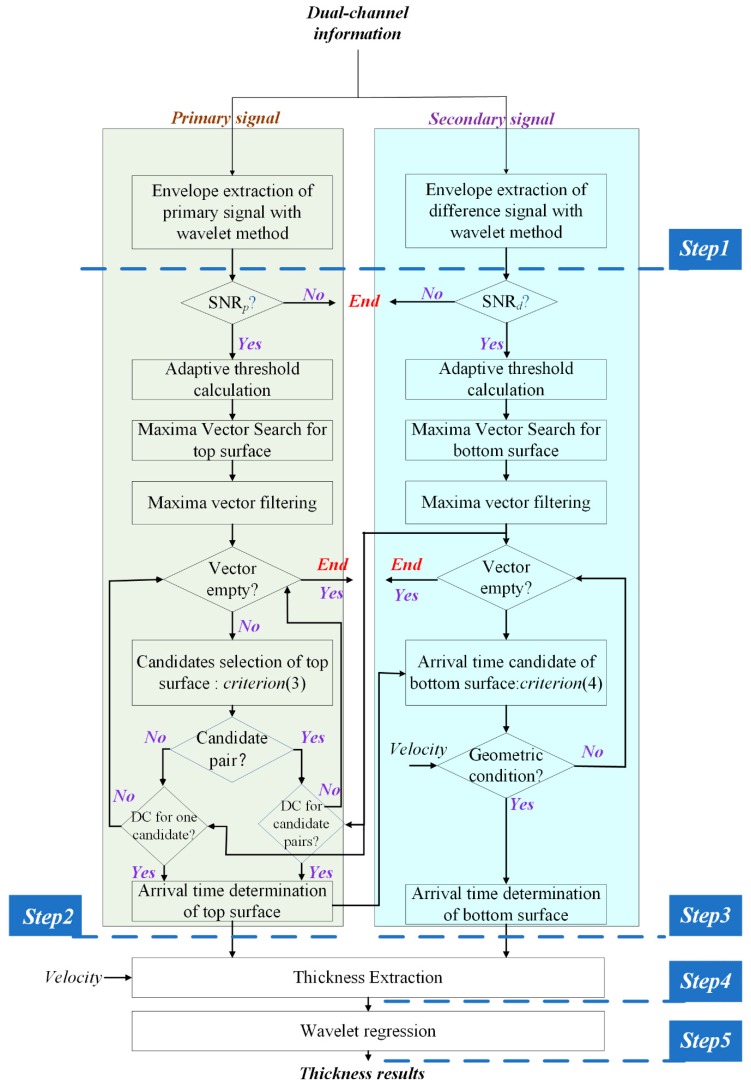
The flowchart of the processing algorithm.

The aim of the algorithm is to automatically calculate the thickness of the CRCs with dual-channel information, assuming that the CRCs are with the structure of the multilayers at the top surface and only one single layer at the bottom surface. For this algorithm, it is demonstrated to handle the case of two layers at the top surface, while the case of three can be deduced in the same way. The CRCs with more than three layers at the top surface are rare. The processing method, as illustrated in Figure 3, is developed to extract the thickness of the CRCs. The algorithm mainly contains five steps based on the integrated utilization of the dual-channel information, i.e., primary signal $S_{p}\left(t\right)$ and the secondary signal $S_{d}\left(t\right)$. The main steps are listed as follows:

*Step 1*. Extract the envelope of $S_{p}\left(t\right)$ and that of $S_{d}\left(t\right)$ with the wavelet method to obtain $W\left(S_{p}\left(t\right)\right)$ and $W\left(S_{d}\left(t\right)\right)$, respectively.

*Step 2*. Determine the arrival time of the top surface with the auxiliary information of the secondary channel. The central idea of this step is that the top arrival time is calculated by using the primary signal with the help of the secondary signal. Such a method has some advantages. Firstly, compared with the secondary signal, the reflected primary signal of the top surface has a relatively higher SNR since the received power of the primary signal is much larger than the secondary signal. Moreover, the resolution of the primary signal is also finer than the secondary signal. The reason that we could use the secondary signal for reference is that the reflection will happen simultaneously on dual-channel. Another important consideration is based on the modeling of the structure of the CRCs. Multiple reflections will occur within the top surface, thus leading to multiple peaks appearing in the primary envelope. The energy of the peaks varies even in the same position which is impacted by the environment, which contributes to inaccuracy in the arrival time estimation. We give a simple way to handle this problem with robust performance.

Specifically, as marked yellow in Figure 3, this step contains several sub-steps. Firstly, the algorithm checks whether the SNR of the primary signal *SNR_p_* satisfies the reliable condition to perform further processing:

(1) $$SNR_{p}=\frac{\max\;W\left(S_{p}\left(t\right)\right)}{E\left[W\left(S_{p}\left(t\right)\right)\right]}>ksnr_{p},$$

where max[⋅] denotes calculating the peak value of the signal, E[⋅] denotes the average operator, and the primary signal threshold, $ksnr_{p}$, is a constant larger than one. Secondly, the primary adaptive threshold can be set to the maximum value according to:

(2) $$TH_{p}=k_{p}\max\;W\left(S_{p}\left(t\right)\right),$$

where $k_{p}$ is a constant less than one Next, the maxima vector search for the top surface, which contains all the candidates of arrival time value, needs to be done. The window is used to partition the signal into some parts, whose size can be empirically set as 200 μs. By using the thresholds (2), the maxima vector is masked by removing the points that are lower than the threshold to reduce the number of candidates. If the masked vector with the length of n, $N_{p}=[N_{1},N_{2},\ldots ,N_{n}]$ is not empty, we can select the candidates based on some criterion. One typical criterion is using the descending selection, i.e., choosing the current maximum from the masked maxima vector first, and the new maxima vector can be given as

(3) $$Ndes_{p}^{}=descend\left\{\left[N_{1},N_{2},\ldots ,N_{n}\right]\right\},$$

The next step is to sequentially fetch two adjacent candidates from the candidate vector:

(4) $$\left(Ndes_{p}^{i},Ndes_{p}^{i+1}\right)\in Ndes_{p}^{},1\leq i\leq n-1,$$

They can be called candidate pair if they satisfy the envelope and the arriving time constraints:

(5) $$20\log_{10}\left(\frac{W\left(Ndes_{p}^{i}\right)}{W\left(Ndes_{p}^{i+1}\right)}\right)\leq 3\;dB,$$

(6) $$\left|Ndes_{p}^{i}-Ndes_{p}^{i+1}\right|\leq TH_{N},$$

where $TH_{N}$ denotes the resolution parameter and can be set according to the resolution. Regardless of the result of the candidate pair or one candidate, the next step is to judge whether the selected point $Ndes_{p}^{i}\in Ndes_{p}^{}$ satisfies the dual-channel constraints (DC) by using:

(7) $$\frac{N_{d}^{j}}{fsL}-\frac{Ndes_{p}^{i}}{fsH}<TH_{\Delta t},for\;all\;N_{d}^{j}\in N_{d},$$

where $N_{d}^{j}$ denotes the arbitrary element of the filtered maxima vector $N_{d}$ for secondary channel, fsL and fsH denotes the sampling frequency of the primary channel and secondary channel, respectively, and $TH_{\Delta t}$ denotes the threshold for arrival time secondary for dual-channel. In other words, Equation (7) exploits the principle of almost simultaneous reflection of the primary signal and secondary signal at the top surface. The division by the sampling frequency converts the discrete number to the arrival time. If the selected point satisfies the constraints, the arrival time of the top surface $t^{top}$ will be determined. Otherwise, the algorithm removes it and continues to go through all the elements of the new maxima vector.

If the estimation satisfies the dual-channel condition, we can determine the arrival time by different cases as:

(8) $$t^{top}=\left\{ \begin{array}{l} t_{i+1}^{top},t_{i}^{top}and\;t_{i+1}^{top}\;exist\;for\;candidate\;pair\\ t_{i}^{top},\;t_{i+1}^{top}\;does\;not\;exist\;for\;candidate\;pair\\ \;or\;for\;only\;one\;candidate\; \end{array}\right.,$$

The reason behind Equation (8) is that we need to solve the ambiguity of the reflection of the multiple layers that occurred at the surface of the CRCs. If we know that two reflections with similar energy happen at the top surface, we will take the arrival time of the latest one as the estimation.

If only one reflection exists or two reflections with the very different energy, we will only take the estimation of the only candidate’s arrival time of the top surface.

*Step 3*. Determine the arrival time of the bottom surface with the auxiliary information of the secondary channel. Firstly, similar to the processing of the primary channel, the algorithm checks whether the SNR of the secondary signal $SNR_{d}$ satisfies the reliable condition to perform further processing with the expression:

(9) $$\frac{\max\;W\left(S_{d}\left(t\right)\right)}{E\left[W\left(S_{d}\left(t\right)\right)\right]}>ksnr_{d},$$

where $ksnr_{d}$ is a constant larger than one. Secondly, the adaptive threshold can be set to the maximum value according to:

(10) $$TH_{d}=k_{d}\max\;W\left(S_{d}\left(t\right)\right),$$

where $k_{d}$ is a constant less than one. Next, the following steps, i.e., maxima vector search for bottom surface, maxima vector filtering, and selection of the arrival time candidate, are performed to determine the arrival time of the bottom surface. They are very similar to the processing of the primary channel. Note that the maxima vector filtering for secondary signal also utilizes the arrival time of the top surface as a reference. After such steps, we can get the bottom arrival candidates $Ndes_{d}^{}$ with the criterion of descending.

It is worth noting that in such steps we exploit the geometric condition to reduce the interferences. The geometric condition is based on the fact that the CRCs of the minimum thickness of 30 mm, $d_{\min}$, can be of most interest as a potential mineral resource, and those of the maximum thickness of 350 mm, $d_{\max}$, can be practically found. In other words, the arrival time candidate of the bottom surface:

(11) $$\frac{\bar{c}}{2}\left(\frac{N_{d}^{j}}{fsL}-t^{top}\right)\in \left(d_{\min},d_{\max}\right),for\;all\;N_{d}^{j}\in N_{d},$$

where $\bar{c}$ represents the average sound velocity within the sampling CRC and the factor of two means the two-way traveling time. Finally, we can get the estimation of the arrival time of the bottom surface, $t_{bottom}$.

*Step 4*. Obtain the thickness of CRCs $Y_{i}$ by using the following equation:

(12) $$Y_{i}=\frac{\bar{c}}{2}\left(t_{bottom}-t_{top}\right)$$

By combining every scalar $d_{i}$ measured at time *i*, we can get the thickness vector *Y* by:

(13) $$Y=[d_{1}^{},d_{2}^{},\ldots ,d_{M}],M=F_{w}T$$

where M denotes the total record number, $F_{w}$ denotes the working frequency, and T denotes the measuring duration.

*Step 5*. Remove the spikes that exist in the thickness vector by using the wavelet regression method.

In this study, the details of the key issues will be discussed in the following sections.

### 3.1. Velocity Measurement

Since the sound velocity of CRCs is not a constant, a feasible way is to measure the values of several samples that are growing in a specific area and take the averaged values as the reference velocity. Here, we adopt the insert substitution method to measure the arrival time difference to calculate the sound velocity. The average sound velocity can be calculated by,

(14) $$\bar{c}=1/\left(\frac{1}{c_{0}}+\frac{\Delta t}{L}\right),$$

where $c_{0}$ denotes the underwater sound velocity, L denotes the thickness of the test sample, and Δt denotes the time difference, i.e., the arrival time without inserting the sample minus that after inserting the sample. The sound velocity measurement can be implemented for multiple times and the average velocity is taken as the reference value.

### 3.2. Dual-Channel Information

Compared with other algorithms that only use one-channel information for processing, the proposed algorithm utilizes dual-channel information which can provide additional information to extract the thickness information more precisely and robust. When the system works, the secondary signal often contains the interferences, making it difficult to estimate the reflection time. Compared with the secondary signal, the primary signal with a centroid frequency of about 1 MHz has a better resolution and less interference when it encounters the top surface. The reflection time of the primary signal not only can be used for the final calculation of the thickness but provides important prior information to determine the reflection on the bottom surface. The secondary signal has a great penetrating capability, which is used for determining the reflection at the bottom surface.

### 3.3. Wavelet-Based Envelope Extraction

The envelope extraction is a key step to obtain the top and bottom reflection time. Hilbert transformation is widely used in many applications related to location or distance measurement. However, extracting envelope with Hilbert transformation is limited by several factors. Firstly, it could be only applied to the narrow-band signal. For the thickness extraction application, the filter for secondary signal has configurations of different bandwidth. Secondly, it has the disadvantage of extracting the high-frequency component as a part of the envelope, which further causes spines. Such a phenomenon will largely impact the estimation of the top or bottom arrival time.

Hence, we use the complex continuous wavelet transform for analysis. The complex wavelet families contain Complex Gaussian Wavelets, Complex Morlet Wavelets, Complex Frequency B-Spline Wavelets, and Complex Shannon Wavelets. To obtain the envelope of the signal f(t), we will adopt the Complex Shannon Wavelets with scale and shift parameter, i.e.,

(15) $$\psi_{a,b}\left(x\right)=\sqrt{f_{b}}\sin c\left(\frac{x-b}{a}f_{b}\right)e^{\frac{j2\pi f_{c}\left(x-b\right)}{a}},$$

where $f_{b}$ is the bandwidth parameter, $f_{c}$ is a wavelet center frequency, a is the scale parameter, and b is the shift parameter. Generally, the value of $\left(f_{b},f_{c}\right)$ varies from multiple combinations such as (1,1.5), (1,1), (1,0.5) and so on. In the thickness application, we empirically choose (1,1.5) as the parameters. Considering the computational time, a can be set to be 32 typically. We will apply the complex continuous wavelet transform to the signal, and we will get the following equation:

(16) $$W_{f}\left(a,b\right)=\int_{-\infty }^{\infty }f\left(t\right)\psi_{a,b}\left(x\right)dt=W_{f}^{r}\left(a,b\right)+jW_{f}^{i}\left(a,b\right),$$

where $W_{f}^{r}\left(a,b\right)$ and $W_{f}^{i}\left(a,b\right)$ denote the real and imaginary parts, respectively. Therefore, the envelope $\left|W_{f}\right|$ can be explicitly represented as:

(17) $$\left|W_{f}\right|=\sqrt{{\left(W_{f}^{r}\left(a,b\right)\right)}^{2}+{\left(W_{f}^{i}\left(a,b\right)\right)}^{2}}$$

### 3.4. Wavelet Regression

When we estimate the thickness after obtaining the top surface and bottom surface arrival time, another important step is to remove the spikes that exist in the thickness signal. General wavelet thresholding is not capable of handling such signals which may have a small number of discontinuities. The method of wavelet regression which penalizes lack of smoothness in the reconstruction is very simple but can maintain sharp changes in the function and suppressing noise spikes.

Assume that the thickness vector which is represented as, $Y_{i}=THK_{i}+n_{i},i=1,2,\ldots ,N$, where $THK_{i}$ denotes the extracted thickness, $n_{i}$ denotes the measuring noise, and *N* is the total number of measurements. After wavelet regression, we can get $\hat{T}HK_{i}=a_{i}$. In vector notation, we can get $a=W^{T}TWY,$ where T is the diagonal matrix with diagonal elements given by:

(18) $$T_{i,i}=\left\{ \begin{array}{l} 1,i\mathrm{th}\;\mathrm{coefficient}\;\mathrm{kept}\\ 0,i\mathrm{th}\;\mathrm{coefficient}\;\mathrm{removed} \end{array}\right.,$$

and W is the discrete wavelet transform matrix. The purpose of the regression is to establish the zeros of the diagonal of T. Then we can build and minimize the proper function to determine the form of T [21]. For example, we can penalize the residual sum of squares and the second derivative of the estimator. After wavelet regression, we can get a good thickness estimation that exhibits good properties in terms of both fit and smoothness, while the sharp changes still retain well.

## 4. Experimental Results and Analysis

### 4.1. Laboratory Experiment with the Phantom

**Figure 4:**
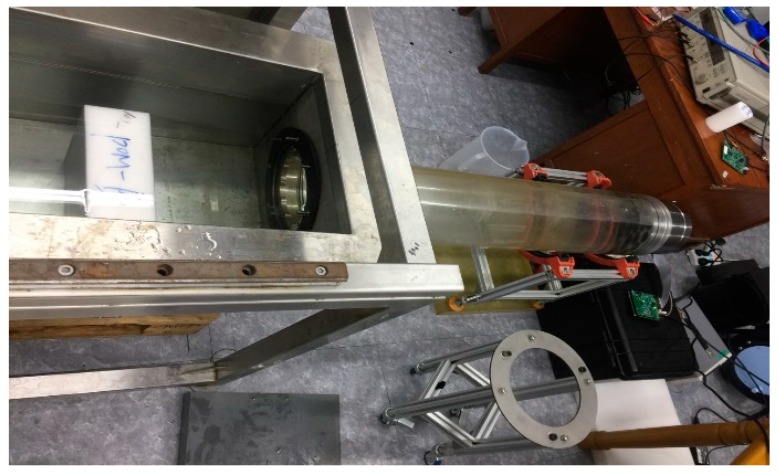
The configuration of the laboratory measurement with the phantom.

To systematically evaluate the effectiveness of the method, a laboratory experiment for measuring a phantom of known thickness was implemented. The experiment configuration is presented in Figure 4. The phantom of the POM material with the thickness of 100.00 mm is placed 1.30 m distance away from the transmitting transducer array.

**Figure 5:**
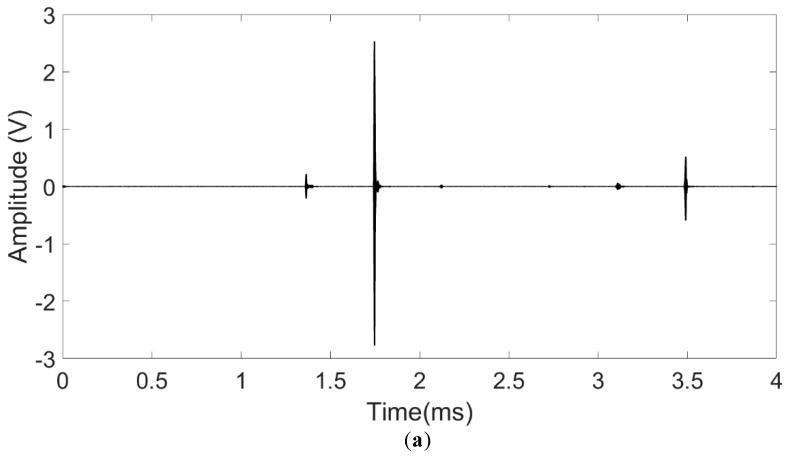

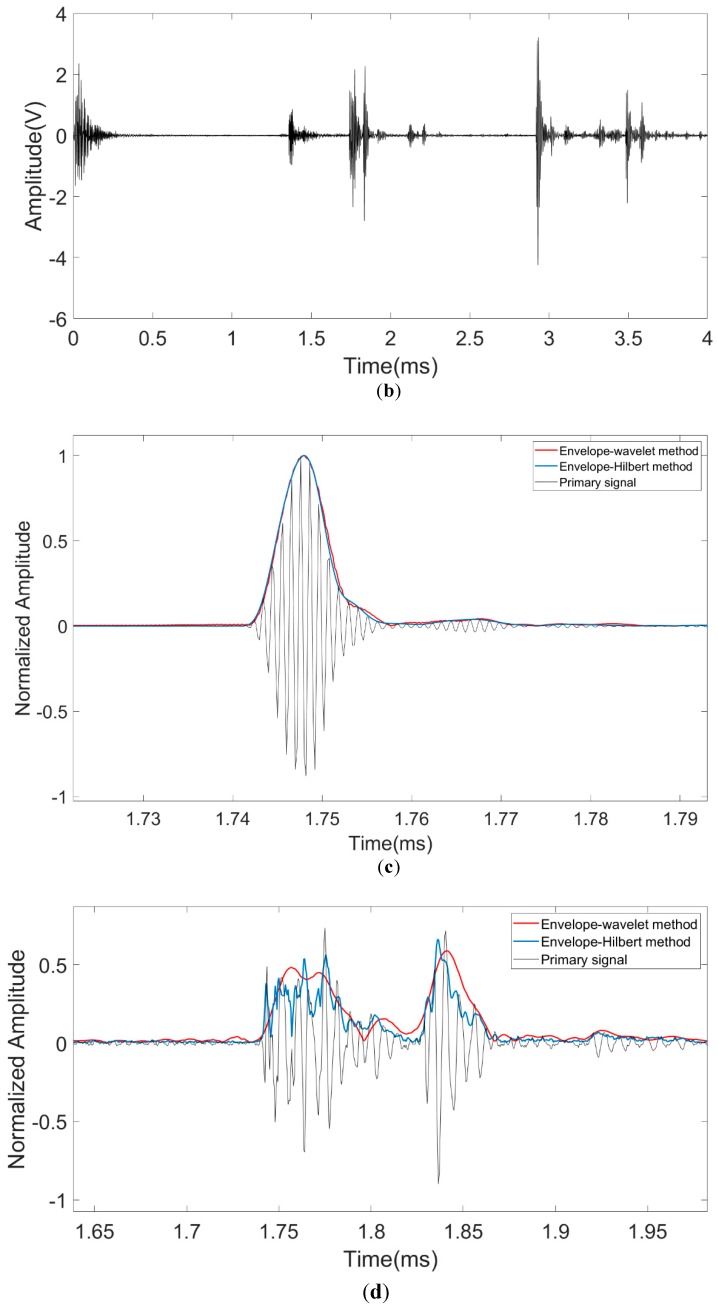
The dual-channel signal of 100th record and the comparison of different envelope extraction methods. (**a**) The primary signal; (**b**) The secondary signal; (**c**) The comparison of the primary channel; (**d**) The comparison of the secondary channel.

In this experiment, the filter parameter is set to be mode 2. The primary data and secondary data contain 160 records, which means the total measuring time is 3.20 s. Taking the 100th signal of the record for example, the primary and secondary channels, as shown in Figure 5a,b, are of good quality with the maximum amplitude value of 2.53 V and 2.93 V, respectively.

Note that the comparisons of the wavelet method and Hilbert method for the dual-channel are given in Figure 5c,d, respectively. Considering that the primary channel has a relatively narrow bandwidth, the difference is not obvious in the waveform although the former has a better quantitative performance than the latter. Figure 5d shows that the envelope extraction of the secondary signal is not robust by the Hilbert method since there are many small high-frequency spikes, while that of the wavelet method is very stable.

The processing parameters are listed in Table 2. The SNR of the primary signal is 50.46 dB, and it is much higher than $ksnr_{p}$. After performing the adaptive threshold masking and the priority calculation with the criterion of amplitude descending, the new maxima vector of the length of five can be obtained as: $Ndes_{p}^{}=[8740,17453,6824,8837,15546]$. Taking the first candidate for example, it is processed as one candidate since it does not satisfy the condition of the candidate pair. The next step is to judge whether the selected point $Ndes_{p}^{i}\in Ndes_{p}^{}$ satisfies the dual-channel constraints by Equation (7). By comparing the real arrival time difference of 9 μs with the threshold $TH_{\Delta t}$, we can verify that the first candidate satisfies the constraints and the value of $t^{top}$ is obtained as 1748.0000 ms.

The SNR of the secondary signal is 44.13 dB, and it is much higher than $ksnr_{n}$. After performing the adaptive threshold masking, maxima vector filtering with the reference of the top surface, and the priority calculation with the criterion of amplitude descending, the new maxima vector can be obtained as: $Ndes_{d}^{}=[5868,3682,3680,3513,3521,6998,5840,7174,6035,7201,5821,6657]$.

Taking the first candidate for example, it is removed since the calculated thickness of 1280.80 mm exceeds the constraints by using Equation (7). By looping the algorithm, we can obtain the second candidate satisfies the constraints and the value of the bottom $t^{bottom}$ is obtained as 1841.0000 ms.

**Figure 6:**
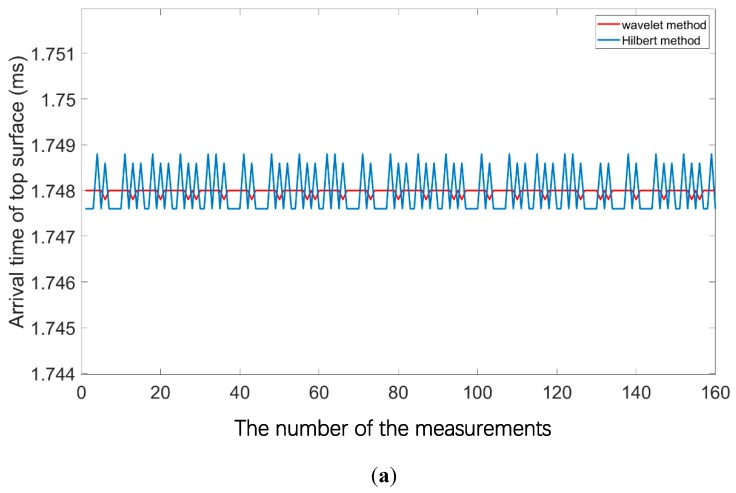

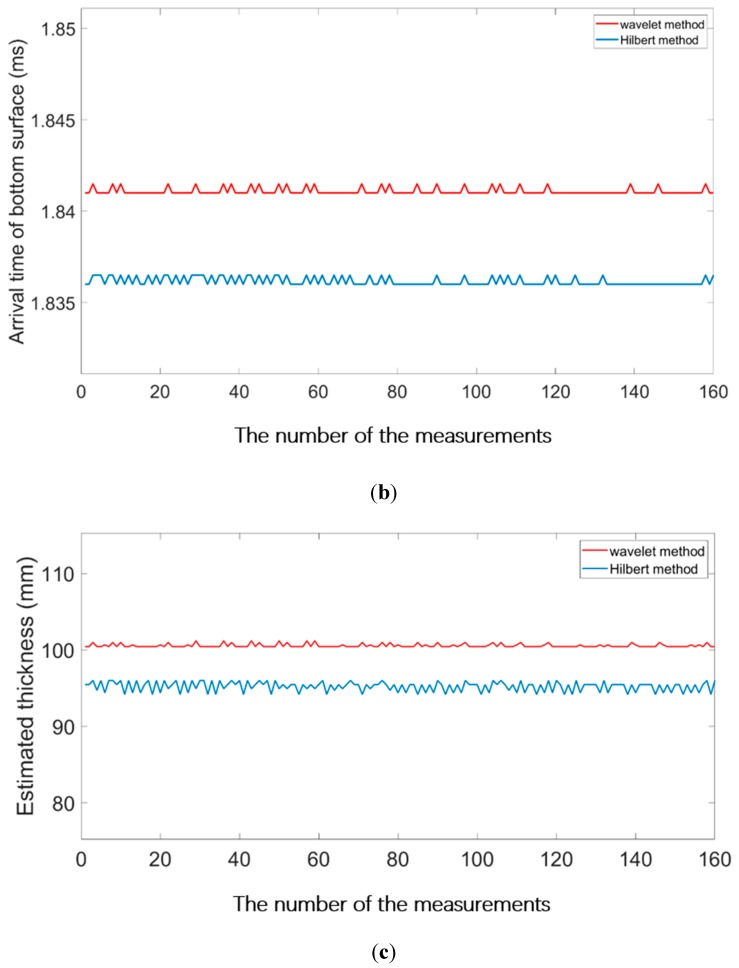
The processing results of the laboratory experiment by different envelope extraction method: (**a**) The arrival time of the top surface; (**b**) The arrival time of the bottom surface; (**c**) The estimated thickness.

The results of the arrival time of the top surface and bottom surface, and the estimated thickness are presented in Figure 6a–c, respectively. The quantitative comparison of the results with the different method is given in Table 3. The sound velocity of the phantom is measured as 2160.0 m/s in the laboratory. It can be seen that the estimated average thickness by wavelet method is closer to the ground-truth thickness of 100.0 mm and the standard deviation is smaller. Next, we use the Haar wavelet of level 6 for wavelet regression. The regression estimate result is 100.55 mm for the Wavelet method, while that is 95.18 mm for the Hilbert method. Since the laboratory experimental environment is good, the improvement of the regressed result is not very obvious. The final obtained results based on the 160 repeated measurements verify the effectiveness of the algorithm.

### 4.2. The Real Experiment in the Western Pacific Ocean

The experiments were carried out on the China Ocean 51th voyage in the Western Pacific Ocean on Aug 30, 2018. The sea-trials were performed in the Western Pacific Ocean with the *sit-on-bottom* stationary measurements aiming to investigate the performance of the acoustic probe and the algorithm. As shown in Table 4, during the survey several measurements were taken at station 10 in the CRCs mountain regions of the interest. As shown in Table 4, we take the data of S1 for example to demonstrate the data process and analysis.

**Figure 7:**
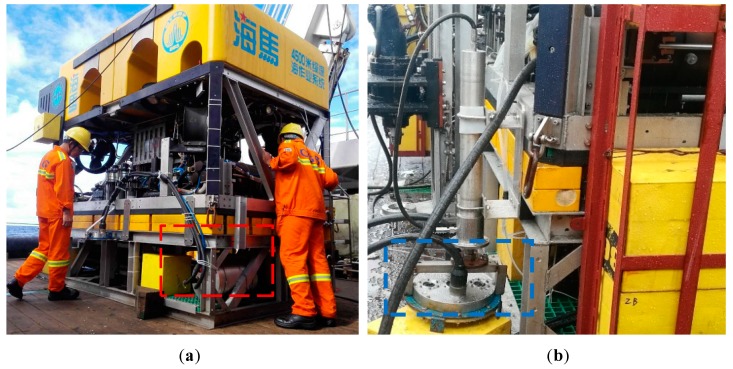
The experimental configuration of PPPAAP17: (**a**) The electronic unit; (**b**) The transmitter transducer array and receiving transducer array mounted on the hydraulic system of the HAIMA ROV.

A brief explanation of the measuring way, i.e., the *sit-on-bottom* stationary measurement, is given here. When performing the *sit-on-bottom* stationary measurement, HAIMA ROV lands fixed at the shoulder of the CRC mountain. As shown in Figure 7a, the electronic processing unit of PPPAAP17 marked with a red dashed box lies at the bottom of the HAIMA Remotely Operated Vehicle (ROV). The transmitter transducer array and receiving transducer array of PPPAAP17 marked with a blue dashed box is mounted on the hydraulic system of HAIMA ROV, as shown in Figure 7b. It can be seen that the vertical distance from the array to the ground could be adjusted. For the stationary measurement, the transmitter transducer array and receiving transducers mounted on HAIMA ROV are about 300 mm away from the seabed in the vertical direction.

The parametric acoustic system, PPPAAP17, transmits the signal for a fixed spot every 20 ms and it samples and keeps the first 2 ms data for each pulse. The data of the primary channel and the secondary channel were separately received and stored. It should be mentioned that the transducer of the secondary channel outstretches 20 mm compared with the primary one, which makes the offset for the reflection instant of the dual-channel. Such a feature causes a fixed time delay difference for the dual-channel when receiving the acoustic echo signal, which could be assumed as the system error. It could be measured in advance and then compensated in the algorithm.

**Figure 8:**
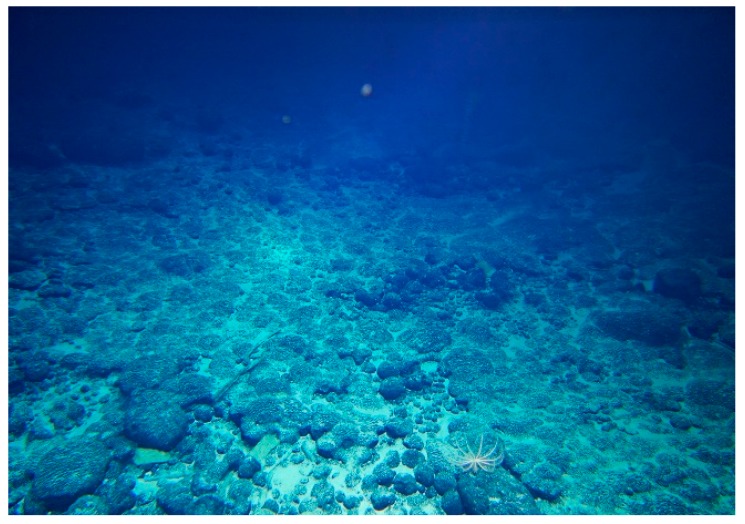
The real scene of the CRCs undersea captured by the underwater camera.

The feasibility of the proposed method and the comparison with the reference method [4] will be given by processing the data acquired by the one stationary measurements, i.e., *Sit-on-bottom 1*(S1). The real scene captured by the underwater camera is given in Figure 8.

**Figure 9:**
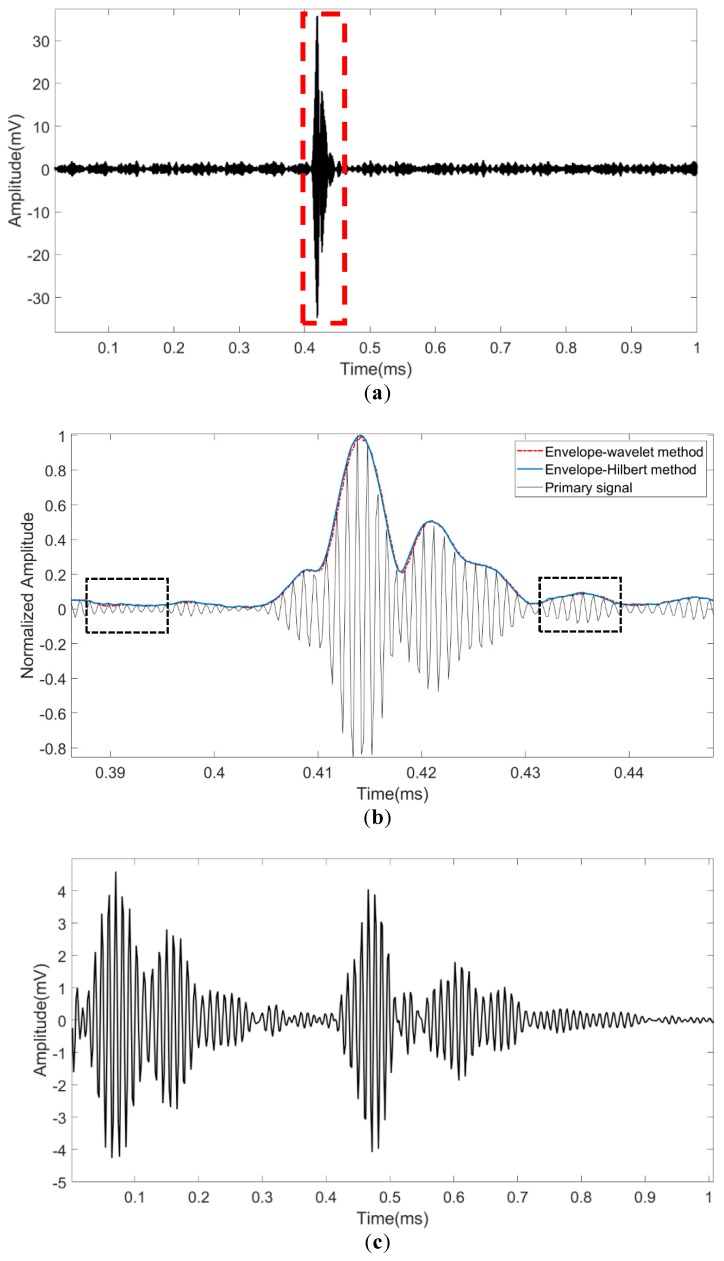

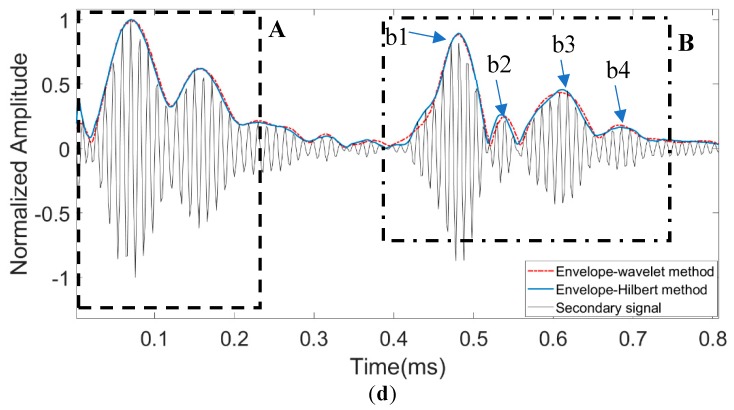
The signal of the 500th record of S1: (**a**) The primary signal; (**b**) The zoomed primary signal with the envelopes extracted by different methods; (**c**) The secondary signal; (**d**) The zoomed secondary signal with the envelopes extracted by different methods with interference.

We select 500 records of the primary data and secondary data, whose duration time is 10.0 s. To better illustrate the features of the reflected signal, we take the 500th primary signal for depiction. As shown in Figure 9a, the primary signal has a good SNR value of 24.88 dB. The signal captured with the red dashed box contains the reflection instant of the top surface. As shown in Figure 9b, the normalized primary signal is zoomed with the extracted envelopes by different methods. Although not very obvious, it still can be seen that the envelope extracted with the wavelet method is better than the Hilbert method at the aspect of the robustness when the high-frequency distortion exists from the areas marked with the dotted box. In addition, we can see that two peaks of the envelope appear at the time of 0.4192 ms and 0.4266 ms, respectively, which means that below the top surface exists another layer. The phenomenon corresponds to the multilayer on the top surface. Figure 9c reveals that the secondary signal also has a good SNR value of 31.13 dB, while the signal has obvious interference which occurs at a very short time after transmission. The interference is marked with the dashed box (A) and the two major reflections are surrounded by the dashed box (B), as shown in Figure 9d.

The first peak (b1) marked with an arrow corresponds to the top reflection and the second peak (b2) is the corresponding tail that is composed of many scattered waves within the footprint when the transmitting wave arrives at the surface. The third peak (b3) and the third peak (b4) correspond to the bottom reflection and scattering, respectively. It is worth noting that the time difference between the first reflection of the dual-channel, which corresponds to the fixed offset and will be compensated at the final step for thickness estimation. Compared with the primary channel, the secondary channel only has one peak at the top surface since the low-frequency envelope has a stronger penetrating capability.

**Figure 10:**
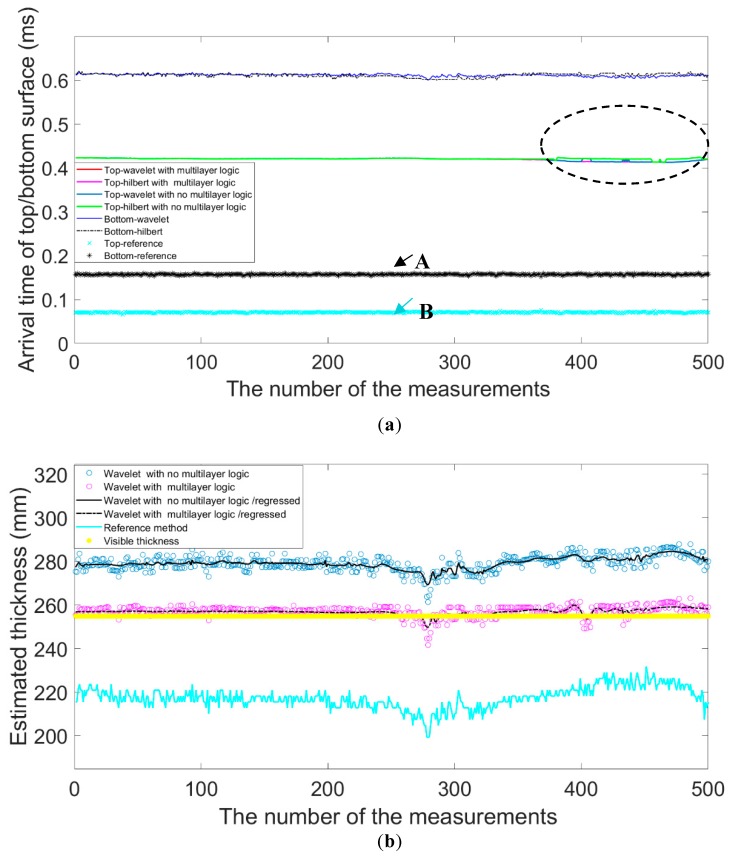
The results: (**a**) The arrival time of the top and bottom surface; (**b**) The estimated thickness by different methods.

To compare the performance of different methods, we present the results of the arrival time of top and bottom surface and the estimated thickness in Figure 10a,b, respectively. As shown in Figure 10a, the two lines marked with A and B denotes the results obtained by the reference method. Due to the fact that the signal is severely interfered, the simple peak search algorithm improperly takes the first two peaks of relatively large amplitude. The proposed method is more robust even when the interference occurs. Besides, as marked by the black dashed circle, it is very obvious that the proposed method has more stable results than others with no multilayer logic.

The comparisons of estimated results and the visible thickness of the CRCs are drawn in Figure 10b. It is worth noting that the estimation of the methods with no multilayer logic has biases than the others since they always extract the first peak that occurred at the top surface. By calculation, we have known that in the presence of noise the reference method could only obtain the wrong estimation which exceeds 500mm. Before we could perform the comparison, the interference is first removed. As shown in Figure 10b, we could obtain the estimation of the reference method is about 220 mm.

To quantitatively assess the results, we list the statistics in Table 5. It can be seen that the simple method with Hilbert/No Multilayer/has a large bias of 11.40 mm and a standard deviation of 17.30 mm given that the visible thickness (#) of 255.00 mm. In contrast, using the simple method with Hilbert/No Multilayer/R we could optimize the stability and obtain the estimation with the standard deviation of 1.23mm. We have known that wavelet regression can optimize the results of all methods. As for the reference method, the estimation has the largest bias and also a large standard deviation, suggesting that it fails to resolve the multilayer case and only achieves the poor precision based on only one channel. Finally, we could see that the proposed method with Wavelet/No Multilayer/R obtains the most accurate results with the mean value of 256.89 mm and the standard deviation of 1.20 mm.

After analyzing the results, we can conclude that the proposed method has several advantages over the reference method. Firstly, the envelope extracted with wavelet transform tends to be more robust even when the SNR decreases. Secondly, based on the multilayer model and local continuity assumption, the proposed method could recognize the correct peak of the top surface reflection even though the value of the peaks varies with the underwater circumstance. In contrast, the reference method could only obtain the first refection peak since it exceeds the threshold, thus leading to inaccurate estimation. In addition to this, the proposed method has a better resolution considering that the sampling frequency of the primary channel is much larger than the secondary one. Finally, using the dual-channel information the proposed method could simply remove the interferences while the reference method could not. From multiple aspects, the results have proven the feasibility of the proposed method.

## 5. Conclusions

This paper described the importance of the CRCs as a potential mineral necessity source and the necessity of mapping and estimating the volumetric distribution of deep-sea mineral. The prototype acoustic probe, i.e., PPPAAP17, was described in detail, aiming to collect and process the data both for the thickness measurement and for the material recognition in the future. As one of the highlights, the prototype was designed with the dual-channel for receiving the primary and secondary signal. Considering that the signal quality is degraded by the system interference and ambient noise, the thickness extraction algorithm was proposed with some improvements, such as the wavelet-based envelope extraction method, the adaptive strategy of the determination of the bottom and top surface arrival time based on the dual-channel information, and the wavelet regression to reduce measuring noise. The algorithm is suitable for the assumption that the CRCs are with the structure of the multilayers at the top surface and one single layer at the bottom surface. Such an assumption is reasonable since it is suitable for some of the real scenes of the CRCs. The algorithm considers the connection of the dual-channel signal, i.e., the features of the CRCs will be shared. The wavelet regression considers the fact of the local invariability of a large amount of data. The laboratory experiment has been demonstrated in the tank and has validated the effectiveness of the processing method. Besides this, the real experiment on the China Ocean 51th voyage in the Western Pacific Ocean on Aug 30, 2018, was carried out by using the sit-on-bottom stationary measurement and the data were obtained. The processing results have shown that the proposed algorithm has very good performance as a standard variation of 1.20 mm (S1) with wavelet regression. As for further research, we will consider applying the Empirical Mode Decomposition (EMD) method to the problem mentioned above. The EMD method decomposes a signal into modes having physical meaning, and it allows for envelope extraction in each mode separately. Besides, the work could be carried out at the aspect of building the more complex thickness model describing the multilayer feature at the bottom surface.

## Figures and Tables

**Table 1:** The filtering and sampling parameters.

Parameter	Symbol	Value
Centroid frequency of the primary channel	$f_{0H}$	1 MHz
The bandwidth of the primary channel	$BW_{H}$	200 kHz
The sampling frequency of the primary channel	$f_{sH}$	5 MHz
Lower/Higher cut-off frequency of the BPFof the secondary channel [mode 1]	$fl_{L1}$/$fh_{L1}$	90 kHz/110 kHz
The sampling frequency of the secondary channel [mode 1]	$f_{sL1}$	500 kHz
Lower/Higher cut-off frequency of BPF ofthe secondary channel [mode 2]	$fl_{L2}$/$fh_{L2}$	100 kHz/380 kHz
The sampling frequency of the secondary channel [mode 2]	$f_{sL2}$	20 MHz
System transmitting frequency	$F_{W}$	50 Hz

**Table 2:** Parameters for the laboratory measurement with the phantom.

Parameters	Symbols	Value
The primary/secondary signal threshold	$ksnr_{p}$ $/ksnr_{d}$	6 dB/6 dB
The primary/secondary adaptive threshold	$k_{p}$ $/k_{d}$	0.02/0.10
The threshold for the arrival time secondary	$TH_{\Delta t}$	20 μs
The threshold for the candidate pair	$TH_{N}$	150

**Table 3:** Quantitative comparison of the results with different methods.

Results	Wavelet Method Mean/std	Hilbert Method Standard Deviation
Arrival time of top surface	1747.9675 ms/0.07 μs	1747.9925 ms/0.53 μs
Arrival time of bottom surface	1841.0813 ms/0.19 μs	1836.1599 ms/0.23 μs
Estimated thickness	100.56 mm/0.221 mm	95.22 mm/0.651 mm
Regressed thickness	100.55 mm/0.006 mm	95.18 mm/0.017 mm

**Table 4:** The data description of the experiment in the Western Pacific.

Station Name	No.	Start Time (UTC+8)	END TIME
10	Sit-on-bottom 1(S1)	2018-8-30-13-24-22	2018-8-30-13-41-31

**Table 5:** Comparison of the thickness with different methods (S1).

**Thickness**	**Hilbert/No Multilayer**	**Hilbert/Multilayer/R**	**Reference** **Method**	**#Visible**
Mean	266.40 mm	266.43 mm	217.33 mm	255.00 mm
Std.	17.30 mm	1.23 mm	14.93 mm	/
**Thickness**	**Wavelet/No Multilayer**	**Wavelet/Multilayer**	**Wavelet/No Multilayer/R**	*Wavelet/Multilayer/R
Mean	279.11 mm	256.88 mm	279.15 mm	256.89 mm
Std.	18.90 mm	15.10 mm	2.50 mm	1.20 mm
